# Bray-Call Sequences in the Mediterranean Common Bottlenose Dolphin (*Tursiops truncatus*) Acoustic Repertoire

**DOI:** 10.3390/biology11030367

**Published:** 2022-02-25

**Authors:** Daniela Silvia Pace, Carla Tumino, Margherita Silvestri, Giancarlo Giacomini, Giulia Pedrazzi, Gianni Pavan, Elena Papale, Maria Ceraulo, Giuseppa Buscaino, Giandomenico Ardizzone

**Affiliations:** 1 Department of Environmental Biology, Sapienza University of Rome, 00185 Rome, Italy; tumino.1701635@studenti.uniroma1.it (C.T.); giancgiacomini@gmail.com (G.G.); g.pedrazzi06@gmail.com (G.P.); giandomenico.ardizzone@uniroma1.it (G.A.); 2 Department of Environmental and Evolutionary Sciences, University Austral of Chile, Valdivia 5090000, Chile; margherita.silvestri@alumnos.uach.cl; 3 Department of Earth and Environmental Sciences, University of Pavia, 27100 Pavia, Italy; gianni.pavan@unipv.it; 4 Consiglio Nazionale delle Ricerche-Istituto per lo Studio degli Impatti Antropici e Sostenibilità, Campobello di Mazara, 91021 Trapani, Italy; elena.papale@ias.cnr.it (E.P.); maria.ceraulo@ias.cnr.it (M.C.); giuseppa.buscaino@cnr.it (G.B.); 5 Department of Life Sciences and Systems Biology, University of Torino, 10123 Torino, Italy

**Keywords:** acoustic sequences, bray-call, geographical differences, common bottlenose dolphin, *Tursiops truncatus*, Mediterranean Sea

## Abstract

**Simple Summary:**

In the acoustic repertoire of common bottlenose dolphins (*Tursiops truncatus*), Gulps, Grunts, and Squeaks are part of a group of vocalizations called “bray-call” for which little has been previously studied. The name comes from the alternating structure characteristic of a donkey’s bray. Sounds can be of different types at low frequencies and audible to the human hear—of short duration, produced in sequence. The function of these sequences is not clarified yet, and it is not known if they are part of the vocal “catalog” of all the different populations of common bottlenose dolphin at global level. What is certain is that bray-calls are present in two geographical areas of the Mediterranean and that the “Capitoline” individuals (Rome, Tyrrhenian Sea, Italy) emit them with greater frequency and variety than the Sicilian ones (Mazara del Vallo, Sicilian Channel, Italy). A number of 13 different types of sequences have been identified, and only 2 of them are shared between the study areas. For the first time this study identifies variants of the main bray-call elements, highlights the structural complexity of these vocalizations, and suggests addressing future research on the context of emissions and the possible function(s) of such acoustic arrangements.

**Abstract:**

Acoustic sequences are commonly observed in many animal taxa. The vast vocal repertoire of common bottlenose dolphins (*Tursiops truncatus*) also includes sequences of multi-unit rhythmic signals called bray-call which are still poorly documented, both functionally and geographically. This study aimed to (1) describe, classify, and characterize series of bray-call recorded in two sites of the Mediterranean basin (Rome—Tyrrhenian Sea and Mazara del Vallo—Strait of Sicily) and (2) investigate for the existence of possible geographic differences. The acoustic analysis identified 13 different sequence types, only two detected in both study areas. The Sørensen–Dice index revealed a low degree of similarity between the sequence repertoire of the two common bottlenose dolphin sub-populations, with the Tyrrhenian being more diversified and complex than the Sicilian one. The acoustic parameters also showed variability between the study area. Different variants of the main acoustic elements composing the bray-call sequences were detected in the Tyrrhenian Sea only. The Markov-chain model demonstrated that the transition probability between acoustic elements is not uniform, with specific combinations of elements having a higher probability of occurrence. These new findings on common bottlenose dolphin bray-call sequences highlight the structural complexity of these vocalizations and suggest addressing future research on the context of emissions and the possible function(s) of such acoustic arrangements.

## 1. Introduction

Sequences of distinct acoustic elements are a widely spread feature of animal acoustic repertoire. They are used mainly in communication and often contain valuable information beneficial for the receiver [1]. Acoustic sequences can convey evidence of individual identity in many species (e.g., frog and insects [2]; common starling, *Sturnus vulgaris* [3]; grey wolf, *Canis lupus lycaon* [4]; common bottlenose dolphin, *Tursiops truncatus* [5]; long-finned pilot whale, *Globicephala melas* [6]), as well as context-related information, such as resources availability (e.g., chimpanzees, *Pan troglodytes* [7]) or predation risk (e.g., *Marmota* spp. [8]; primates, *Colobus polykomos* and *Colobus guereza* [9]). However, there are many cases in which the ultimate function of the acoustic sequences is still poorly understood.

Since acoustic sequences are composed of distinct elements (i.e., acoustic units [1]), identifying and distinguishing their relevant components is a preliminary step to understand their structure, potential meaning, and possible function. Characterizing a sequence, and its emission context, is crucial to determining which kind of information it may contain and how this information may be encoded in its structure [1]. According to the classification method proposed by [1], information can be codified within the acoustic sequences following six different paradigms: (a) *Repetition*, where a single unit is repeated more than once; (b) *Diversity*, where information is represented by the number of distinct units present; (c) *Combination*, where sets of units have different information from each unit individually; (d) *Ordering*, where the relative position of units to each other is important; (e) *Overlapping*, where information is conveyed in the relationship between sequences of two or more individuals; and (f) *Timing*, where the time gap between units conveys information.

The production of multi-element acoustic sequences is documented in several cetacean species (e.g., sperm whale, *Physeter macrocephalus*: [10,11]; killer whale, *Orcinus orca* [12]; humpback whale, *Megaptera novaeangliae* [13]; long-finned pilot whale, *Globicephala melas* [14]). The complex vocal repertoire of common bottlenose dolphin also includes repeated acoustic sequences such as the signature whistle identity calls [15,16]. Among them, this species emits sequences of multi-unit rhythmic signals known as bray-call series [17,18]. These sequences have been recorded for the first time during social interactions in the Sado estuary, Portugal [19,20] and have been detected occasionally in other populations (e.g., [21,22,23]), also in feeding contexts [24,25]. dos Santos et al. [19] described the bray-call series as sequences of two alternating sounds: a “squeak” (a pulsed sound with a repetition rate of 10–20 click/s, duration ranging 1–2 s and peak frequency ranging 4–6 kHz) and a “grunt” (a low-frequency continuous not pulsed sound), subsequently renamed “gulp” five years later [20]. A similar structure for bray-call sequences was suggested by [24], that described them being composed of a burst-pulsed sound and a short downsweep low-frequency sound. Lately, Janik et al. [17] described the bray-call sequences as composed by a variable number and type of units with three main acoustic elements: “gulp” (impulsive low-frequency sound), “grunt” (series of broadband impulsive signals with most of the energy at low frequencies), and “squeak” (pulsed sound perceived as a tonal sound by the human ear). Three typical and well-defined call arrangements were identified: single-unit gulp or grunt sequences, and multi-unit sequences that contain squeaks [17]. This variability in the composition of the sequences was ascribed to possible different meanings of the units, suggesting the existence of diversity, combination, repetition, time, and order rules [1,17].

Bray-calls are still poorly documented, functionally and geographically [17]. This study aims to provide a detailed structural description, classification, and characterization of the bray-call sequences recorded in two different areas of the Mediterranean Sea (Rome—Tyrrhenian Sea and Mazara del Vallo—Strait of Sicily). In addition, the possible presence of geographical differences between sub-populations within the basin was investigated comparing acoustic data from the two sites.

## 2. Materials and Methods

### 2.1. Study Area

**Figure 1:**
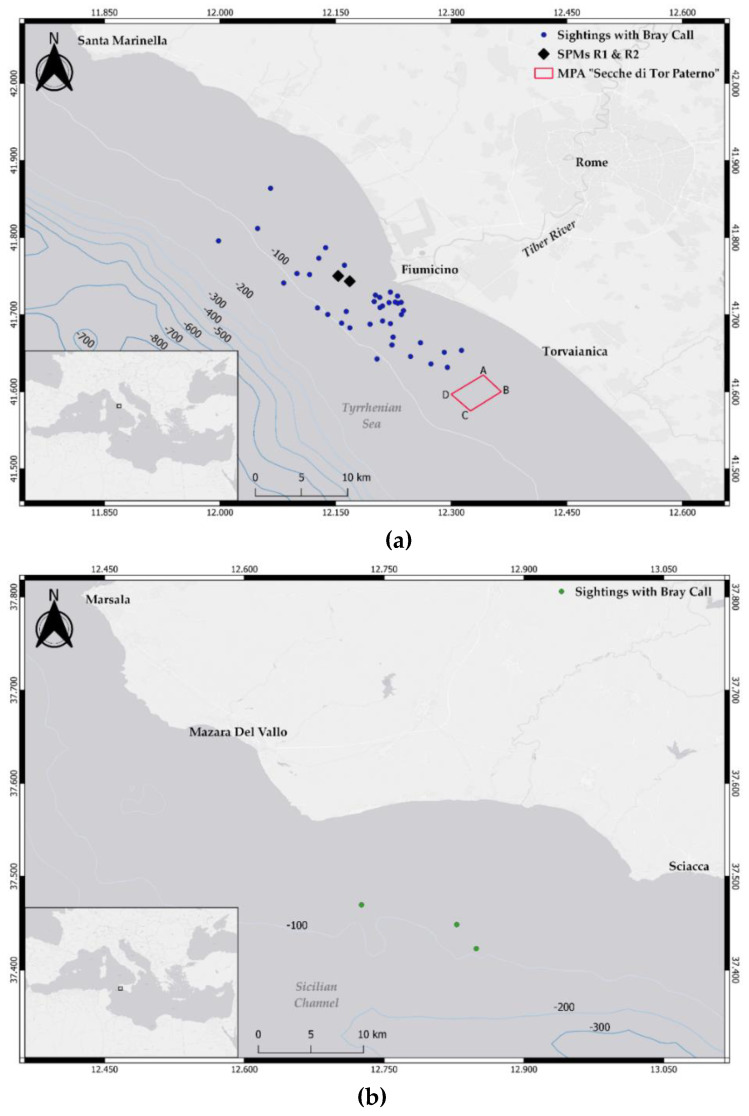
Maps of the two study sites. (**a**) Tyrrhenian Sea. The two black rhombi represent the single point moorings R1 and R2; the red rectangle identifies the “Secche di Tor Paterno” MPA; blue dots show the spatial distribution of the common bottlenose dolphin encounters with bray-call detections. (**b**) Strait of Sicily. The green dots show the spatial distribution of the common bottlenose dolphin encounters with bray-call detections.

Recordings were collected in the central Mediterranean (Rome, Tyrrhenian Sea) and the Strait of Sicily (Mazara del Vallo) (Figure 1).

The Tyrrhenian Sea site covers an area of approximately 1300 km^2^ and includes the estuary of the Tiber River (bottom depth up to 100 m). A variety of habitats and environmental conditions characterize the site, with a rich coastal biodiversity principally maintained by the organic matter transported at sea through the river (for a detailed description of the site see [26]). This area is exposed to intense anthropic activity, with a strong level of vessel traffic generated by both tourism and commercial fishery (artisanal and trawling vessels [26,27]), and the effect [28] of two fixed single-point moorings (SPMs, called R1 and R2) receiving crude oil located three nautical miles away from the river estuary. The regular occurrence of groups of common bottlenose dolphin is here reported [29], with numerous sightings of mother and calf pairs, feeding activities, and significant interactions with fishing gears (both trawls and gillnets), allowing the area being identified as a foraging and nursery ground for the species [26,27].

The Strait of Sicily site divides the Tyrrhenian and the western Mediterranean Sea from the eastern side of the basin. More specifically, the study area covers approximately 1440 km^2^ and is located in the north-western side of the strait, along the southern coast of Sicily, between Mazara del Vallo and Sciacca. This area is characterized by a wide and not very deep continental shelf (Adventure Plateau), with a maximum depth of 200 m, and is exposed to intense vessel traffic, being one of the main routes for commercial shipping and fishery. The regular presence of common bottlenose dolphins has been documented in the area, with frequent interactions between common bottlenose dolphins and fishery [30,31].

### 2.2. Data Collection

Acoustic recordings were collected during boat-based daily surveys in both study sites (Table 1) in different years with different methods and equipment. In the Tyrrhenian Sea the sampling effort was conducted for 4–5 months per year (June–October), while in the Strait of Sicily the sampling effort was conducted for 3 months per year (July–September). When a group of dolphins (i.e., two or more individuals involved in similar behavior within a range of 100 m one from the other; [32]) was sighted, GPS position, time, group size, and composition (based on the classification reported in [26]), predominant behavior (i.e., the behavioral state in which more than half of the individuals within the group are involved; [33]), acoustic emissions, and interaction with fishing gears (if any) were recorded. Photographs of dorsal fins were collected using digital cameras equipped with zoom lens, and then used for identification purposes (details on photo-identification procedures in [26,34]).

### 2.3. Acoustic Analysis

Recordings from the two study sites were first examined to select those containing bray-call series. Visual inspection of the spectrogram was conducted using the software Raven Pro 1.6 [37] with the following settings: Hann window 2048, DFT 2048, Overlap 50%, Hop size 1024. Analysis parameters were optimized to improve signal visualization. The acoustic analysis was conducted on sequences composed by the succession and the combination of acoustic units classified as bray-call elements by [17]: gulp, GU; grunt, GR; and squeak, SQ. The element types were considered as part of the same sequence if the inter-element interval was shorter than 1 min [24]. Other sounds than GU, GR, and SQ were recorded within sequences. These sounds included Pops (POP; low frequency pulsed sound with most energy between 0.3 and 3.0 kHz [38]), Cracks (CR; broadband intense, single clicks with most of energy between 0.1 and 8.0 kHz [18]), and Low-Frequency Narrowband sounds (LFN; short downsweep sounds with fundamental frequency lower than 2 kHz [18]). They were considered part of the bray-call series if detected within the sequence and less than one minute apart from a bray-call element.

**Figure 2:**
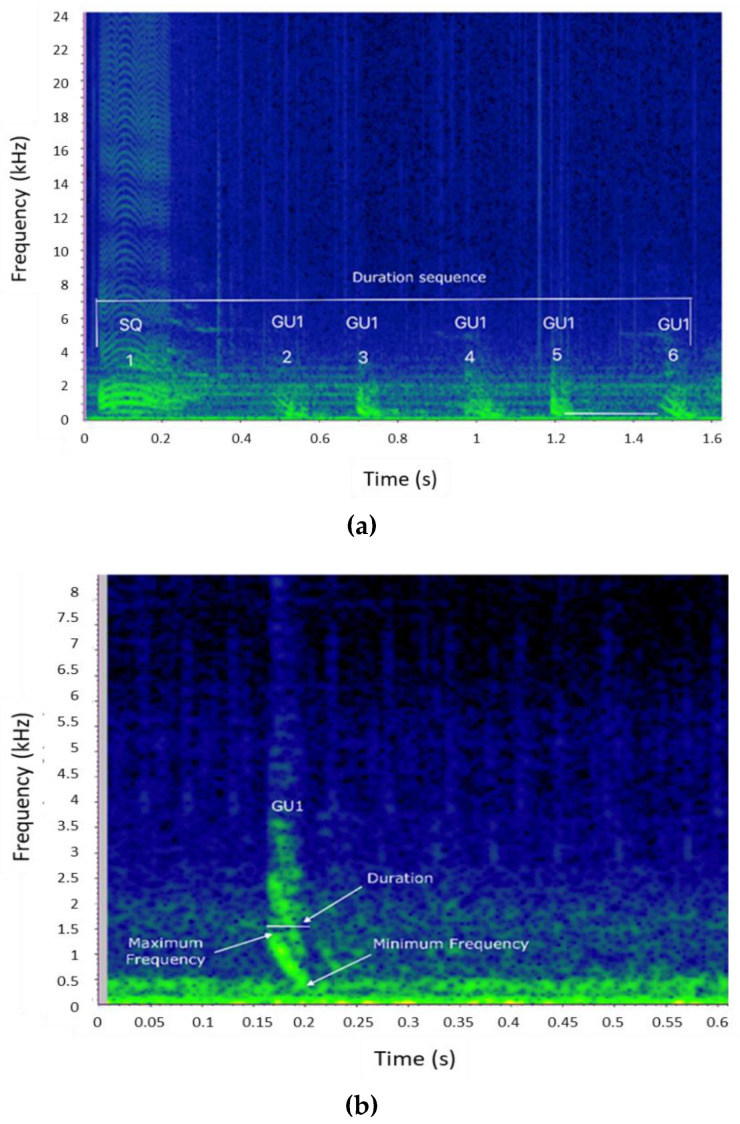
(**a**) Spectrogram of a bray-call series indicating the parameters measured for the sequence (duration, type, and number of elements composing the sequence) and inter-element interval (period) (Hann window, size 2048, DFT 2048, overlap 50%, hop size 1024, sampling frequency 192 kHz, frequency resolution 93.75 Hz, analysis bandwidth 135 Hz at -3 dB). (**b**) Spectrogram of a single Gulp (GU) showing the acoustic parameters extracted for the element (Hann window, size 3000, DFT 4096, overlap 80%, hop size 600, sampling frequency 192 kHz, frequency resolution 46.875 Hz, analysis bandwidth 92.16 Hz at -3 dB).

Each sequence and each element type composing the sequence were characterized by measuring the parameters described in Table 2 and presented in Figure 2. All the variables were extracted using Raven Pro 1.6 and only high-quality sequences (i.e., sequences in which all parameters were measurable) were considered in this work.

**Figure 3:**
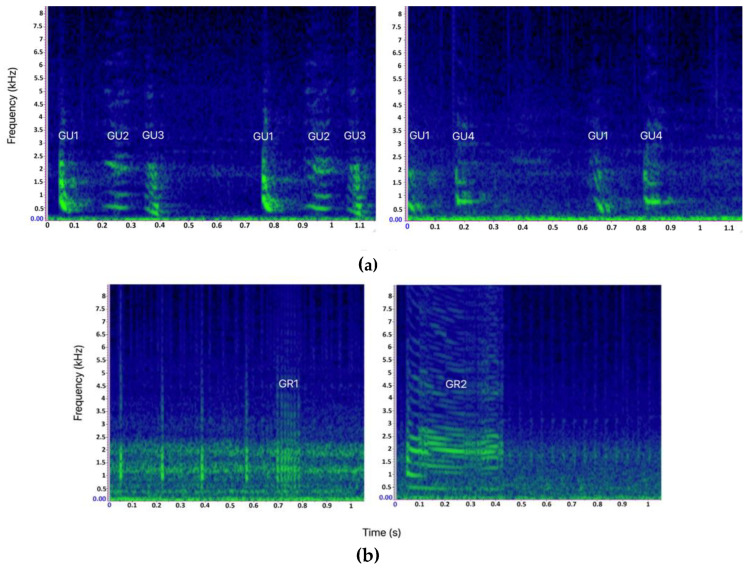
(**a**) Spectrograms of the 4 different Gulp variants (GU1, GU2, GU3, and GU4) identified in this study. (**b**) Spectrograms of the two different Grunt variants (GR1 and GR2) identified in this study (Hann window, size 3000, DFT 4096, overlap 80%, hop size 600, sampling frequency 192 kHz, frequency resolution 46.875 Hz, analysis bandwidth 92.16 Hz at -3 dB).

Main bray-call elements (GU, GR, and SQ) were generally described without intra-type variability. Here, four variants (or “subtypes”) of GU and two variants of GR were identified (Figure 3). A variant was defined as an acoustic unit that can be ascribed to a specific bray-call element type (as described in the literature for its aural and visual characteristics) but showing peculiar acoustic features that allow it to be distinguished from other variants of the same element type. The variants were categorized assigning an identification code composed of the abbreviation of the corresponding element type followed by a number (e.g., the first GU variant was coded as GU1). In this way, it was possible to classify each sequence by the pattern of succession and/or combination of the types/variants of the acoustic elements, and apply the paradigms proposed by [1] for encoding information.

### 2.4. Descriptive and Statistical Analysis

The structural and temporal characteristics of the bray-call series were assessed using a first-order Markov chain model (FOMM) [39]. To evaluate the structural complexity within these bray-call series, Shannon entropic orders [40] were calculated as:

$$H_{1}=\sum_{i=1}^{N}pi\;log_{2\;}pi$$

where *N* is the number of different acoustic elements and *pi* is the probability of the *i*-th acoustic element in a bray-calls series. The FOMM was first applied to the acoustic elements identified as bray-call in the literature (GU, GR, and SQ) [17] and then repeated considering the different variants identified for the first time in this study.

The acoustic parameters extracted from the sequences found in both study areas were compared to investigate for any geographical difference. All continuous quantitative variables were tested for normality distribution through the Shapiro–Wilk test. In case of normal distribution, Welch’s *t*-test were applied, while non-normally distributed variables were tested through the Mann–Whitney and Kruskal–Wallis tests. An index derived from the Sørensen–Dice coefficient of association (SDC) was applied to obtain a measure of the similarity between the bray-call sequence repertoires of the two common bottlenose dolphin sub-populations. The index was computed from the degree of sequence typologies and acoustic element types shared and accounts for differences in the repertoire size. The Sørensen–Dice index was calculated as

SDC = 2 (Ns + Ne)/(R1 + R2)

where Ns is the total number of sequences shared, Ne is the total number of acoustic elements shared, and R1 and R2 are the repertoire sizes of the two sub-populations (distinct sequences + acoustic elements). All analysis were performed in R 4.0.3 (www.r-project.org; accessed 18 December 2021).

## 3. Results

**Figure 4:**
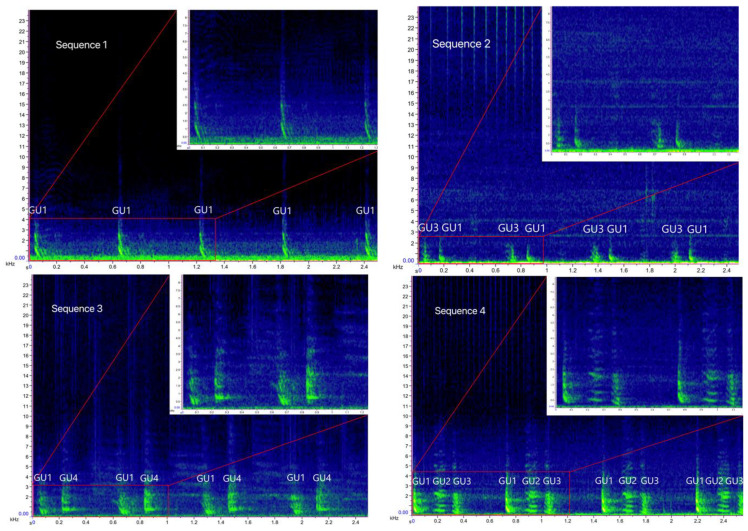

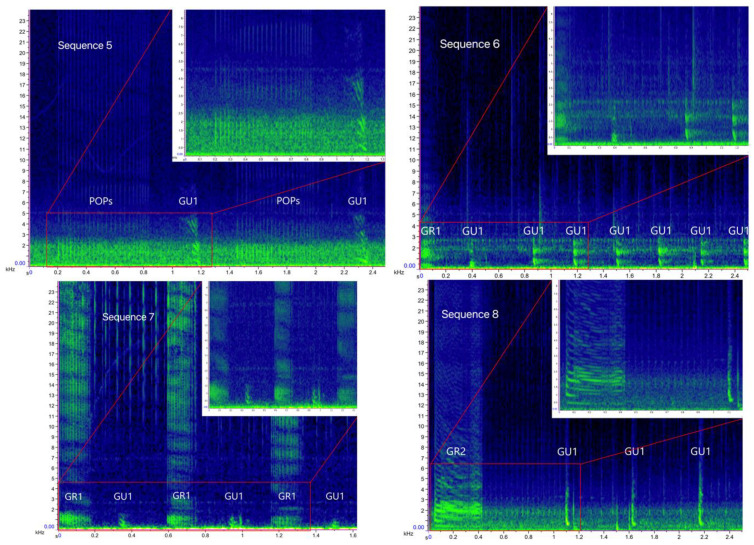
Spectrograms of the bray-call sequences 1–8 identified in the present study (main panels: Hann window, size 2048, DFT 2048, overlap 50%, hop size 1024, sampling frequency 192 kHz, frequency resolution 93.75 Hz, analysis bandwidth 135 Hz at -3 dB); zoomed panels: Hann window, size 3000, DFT 4096, overlap 80%, hop size 600, sampling frequency 192 kHz, frequency resolution 46.875 Hz, analysis bandwidth 92.16 Hz at -3 dB).

**Figure 5:**
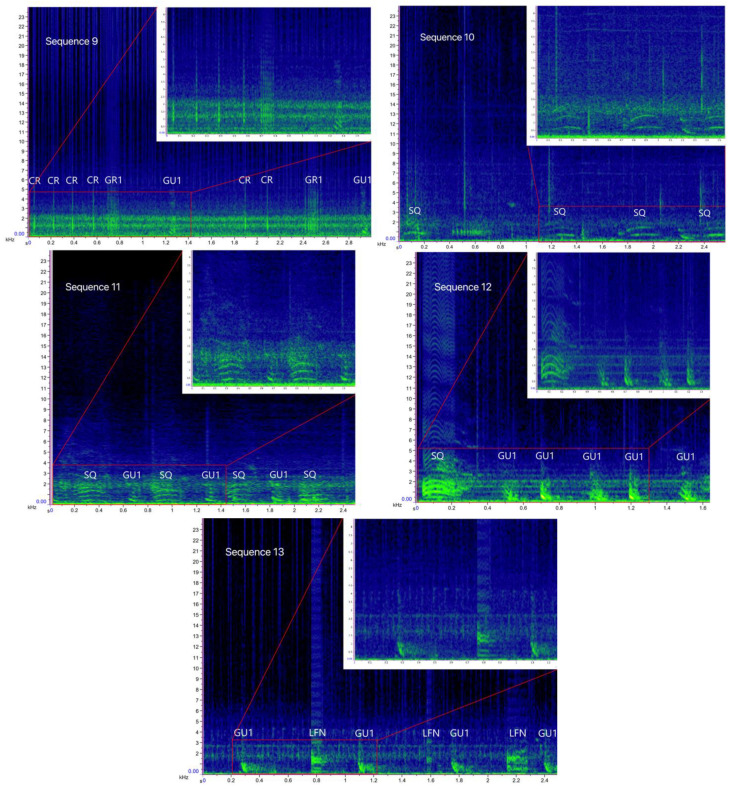
Spectrograms of the bray-call sequences 9–13 identified in the present study (main panels: Hann window, size 2048, DFT 2048, overlap 50%, hop size 1024, sampling frequency 192 kHz, frequency resolution 93.75 Hz, analysis bandwidth 135 Hz at -3 dB); zoomed panels: Hann window, size 3000, DFT 4096, overlap 80%, hop size 600, sampling frequency 192 kHz, frequency resolution 46.875 Hz, analysis bandwidth 92.16 Hz at -3 dB).

**Figure 6:**
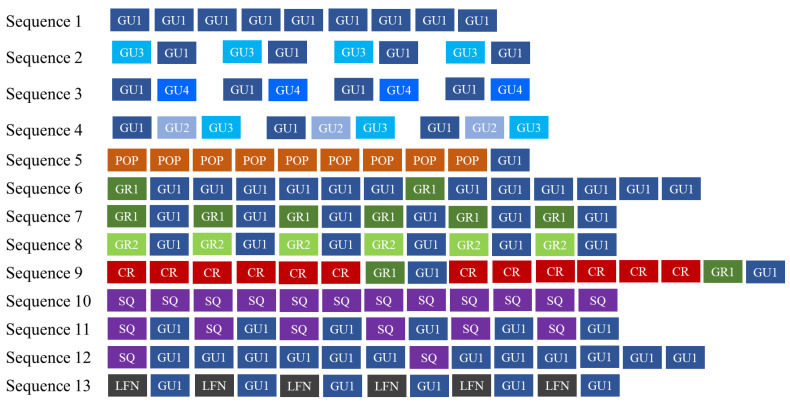
Bray-call sequences identified in this study met five distinct paradigms suggested by [1] for information encoding (repetition, diversity, combination, ordering, and timing).

Overall, 1293 .wav files, corresponding to 119 h of recordings, were examined, 134 of which containing bray-call series. A total of 644 high-quality bray-call sequences including 4030 acoustic elements were analyzed, with 637 in the Tyrrhenian Sea (0.09 sequence/min) and 7 in the Strait of Sicily (0.03 sequence/min). A total of 13 different typologies of stereotyped sequences were identified (Table 3, Figure 4 and Figure 5), all of them detected in the Tyrrhenian Sea and only two in the Strait of Sicily. These bray-call series met five distinct paradigms suggested by [1] for information encoding (repetition, diversity, combination, ordering, and timing; Figure 6). Although more than one animal was spotted during recordings, overlapping between different sequences was never observed.

Sequence 1 accounted for 53.4% in the Tyrrhenian Sea (*n* = 340), followed by sequence 2 (*n* = 78), sequence 9 (*n* = 36), sequences 5 and 10 (*n* = 27 and *n* = 26 respectively), and sequences 7 and 8 (*n* = 25 each); the other five sequences were about 2.5% each. In the Strait of Sicily, only single-element sequence 1 (85.7%) and 10 (14.3%) were detected. Indeed, the Sørensen–Dice index revealed a low degree of similarity between the sequence repertoire of the two common bottlenose dolphin populations (similarity, SDC = 0.27), with the Tyrrhenian being more diversified and complex than the Sicilian one.

As shown in Table 3, the primary element type composing the sequences was Gulp (80%). The variant GU1, present in all multi-element sequences, had down-sweep contour, no harmonic-like structure, and a wider frequency range than all other Gulp variants (minimum frequency = 295 ± 94 Hz, CI = 291–299 Hz; maximum frequency = 773 ± 61 Hz, CI = 766–779 Hz; duration = 0.04 ± 0.01 s, CI = 0 s). The variant GU2 had a mostly flat shape and a harmonic-like structure (minimum frequency = 361 ± 83 Hz, CI = 345–378 Hz; maximum frequency = 584 ± 70 Hz, CI = 570–598 Hz; duration = 0.04 ± 0.01 s, CI = 0.04–0.07 s). As GU1, the variant GU3 had a down-sweep contour, but presented a harmonic-like structure and lower minimum and maximum frequency (minimum frequency = 2168 ± 45 Hz, CI = 210–221 Hz; maximum frequency = 541 ± 78 Hz, CI = 538–550 Hz; duration = 0.04 s, CI = 0 s). As GU2, the variant GU4 also had a mostly flat shape, but without a harmonic structure, and higher minimum and maximum frequency than all other Gulp variants (minimum frequency = 592 ± 71 Hz, CI = 588–597 Hz; maximum frequency = 868 ± 86 Hz, CI = 851–886 Hz; duration = 0.07 ± 0.06 s, CI = 0.03–0.06 s). The element type Grunt was recorded in about 3% of the sequences. The duration of the variant GR1 varied significantly between sequence 6 (0.11 ± 0.3 s), sequence 7 (0.14 ± 0.1 s), and sequence 9 (0.14 ± 0.1 s) (Kruskal–Wallis test, *p* = 0.005); the variant GR2 presented a harmonic-like structure likely due to high click’s repetition rate. The acoustic structure of Squeak (3%), Crack (4%), Pop (10%), and LFN (<1%) was similar to the ones reported by [18].

**Figure 7:**
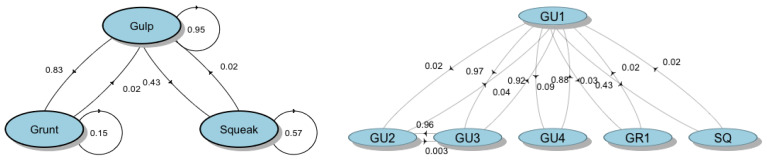
Transition diagrams of bray-call series obtained from first-order Markov chain model. The circles represent different vocal elements, and the values represent the transition probabilities.

The first-order Markov chain model (FOMM) showed that the transition probability between the acoustic element composing the bray-call sequences is not uniform. The highest probability is for a Gulp to be followed by another Gulp (P_GU-GU_ = 0.95), then the probability that a Gulp is followed by a Grunt (P_GU-GR_ = 0.83) and the probability that a Gulp is followed by a Squeak (P_GU-SQ_ = 0.43); single-element sequences of repeated Squeak showed a probability of occurrence of approximately 60% (P_SQ-SQ_ = 0.57) (Figure 7, left side). The FOMM also revealed that there is a very high probability to observe GU1 followed by GU3 (P_GU1-GU3_ = 0.97) and vice-versa (P_GU3-GU1_ = 0.92), GU3 followed by GU2 (P_GU3-GU2_ = 0.96) and GU4 followed by GU1 (P_GU4-GU1_ = 0.88). The GU2, GU3, and GU4 variants have never been observed in sequences with Squeaks or Grunts (Figure 7, right side).

**Figure 8:**
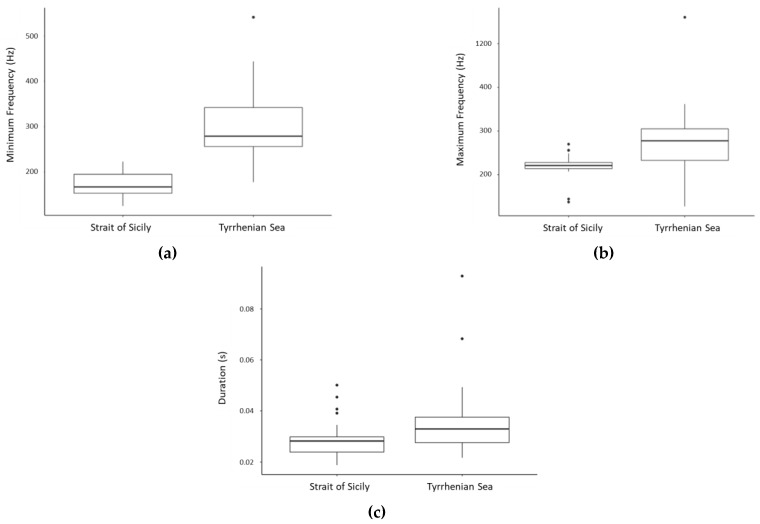
Box plots showing the distribution of minimum frequency (Hz) (**a**), maximum frequency (Hz) (**b**), and duration (s) **(c)** of Sequence 1 in the two study areas.

Average values of the acoustic parameters of the 13 sequences, and of the element types and variants measured in each sequence, are reported in Table 4. Minimum frequency (Welch *t*-test: *t* = −9.4845, df = 48.703, *p* < 0.001), maximum frequency (Mann–Whitney: W = 219, *p* < 0.001) and element duration (Mann–Whitney: W = 288, *p* < 0.05) of Sequence 1 (the most recorded sequence) resulted to vary significantly between study areas, with lower values measured in the Strait of Sicily (Figure 8). No significant difference resulted for period and sequence duration.

## 4. Discussion

Acoustic sequences are common events in many animal taxa [1] and their adaptive role has been intensively studied [41]. In common bottlenose dolphin, the structure, characteristics, and use of bray-call series have been investigated in few geographical areas (Portugal: [17,19,20,42]; Scotland: [24,25]; Gulf of Mexico: [43]; Namibia: [22]) and this is the first study that aimed to provide a detailed acoustic description of bray-call sequences from two different sites of the Mediterranean Sea. Here, three main elements composing the sequences have been identified (Gulp, Grunt, and Squeak), as in other studies [17,24,43]. However, the detailed acoustic analysis of the elements led to the description of 4 Gulp variants, identified for the first time in the present study, 2 Grunt variants and the presence of non-bray elements (POP, Crack, and LFN) within the sequences.

The GU1 variant was the most frequent and has been found in all multi-element sequences, suggesting a possible central role in the bray-call series in both Mediterranean sites. The visual classification and the acoustic features of GU1, GU2, and GU4 variants here described do not match with previous Gulp descriptions found in the literature, whereas the variant GU3 shows a high level of similarity with the Gulp described by [17] when considering minimum frequency, maximum frequency, and duration. The variant GR1 seems like the Grunt described by [17], while the variant GR2 shows similarities with one Grunt category recorded in the Guiana dolphin (*Sotalia guianensis*) [44]. The element SQ appears to have similar acoustic characteristics with the Squeak described by [17] in common bottlenose dolphins off Portugal’s coast. LFN sounds here identified are comparable to [18] but different from [43] as they show higher values of minimum frequency, maximum frequency and duration, even though they share an analogous harmonic structure, and are emitted in series. Finally, in this study CRs are as described by [18] and POPs are similar to [38], who reported that these sounds are emitted in series of 3–30 during agonistic interactions between males. 

Overall, these results seem to suggest that Mediterranean common bottlenose dolphin sub-populations may compose sequence using several different acoustic elements and arrange them in a very flexible and complex way to produce distinct sequence types. Indeed, 13 different sequences have been identified, with a different transition probability among elements composing them (as observed by [17]). Two of them are composed by the repetition of a single acoustic element (GU1 in sequence 1 and SQ in sequence 10). The repetition of the same acoustic element within a sequence is reported as a transversal rule for encoding information in mammals’ communication [45,46] and the number of elements per sequence may influence the information codified in the message [1]. Repetition may be used to confirm [47], substitute or reinforce the message [48]. Indeed, a high repetition rate of the same acoustic signal has been often observed in contexts where the correct transmission of the message is fundamental for survival, as in dangerous (e.g., suricates, *Suricata suricata* [49]; marmots, *Marmota* spp. [8]; colobuses, *Colobus* spp. [50]; Campbell’s monkey, *Cercopithecus campbelli campbelli* [51]; lemurs, *Lemur catta* and *Varecia variegate* [52]) or foraging situations (e.g., big-footed myotis, *Myotis macrodactylus* [53]) in terrestrial mammals, and in noisy environments (e.g., blue whale, *Balaenoptera musculus* [54]; killer whale, *Orcinus orca* [55]) in marine mammals. The multi-element sequences identified in the study include seven typologies characterized by a higher level of diversity, timing, and combination of acoustic elements (Sequences 5, 6, 7, 8, 11, 12, and 13) and four typologies characterized by a specific repetition order of pairs and triads (Sequences 2, 3, 4, and 9). Complex acoustic sequences are parts of the vocal repertoire of many animal species and are used for different purposes: improving the communication efficiency (e.g., colonies of bats, *Pipistrellus pipistrellus* [56]), simplifying individual recognition (e.g., humpback whale, *Megaptera novaeangliae* [13]), rejecting competitors and predators (e.g., Campbell’s monkey, *Cercopithecus campbelli* [57]) and during courtship behavior (e.g., common grackles, *Quiscalus quiscula* [58]). It remains to be investigated if bray-call sequences may play similar roles in common bottlenose dolphin communication. Here, five distinct paradigms for encoding information in sequences (Repetition, Diversity, Combination, Ordering and Timing; [1]) are met, but not overlapping between sequences (likely individuals were not vocalizing at the same time in the analyzed dataset). It was not possible to understand with certainty whether each sequence was only emitted by one animal or whether the sequence was made with the contribution of other animals. Thus, the hypotheses of a sequence emitted by one specimen or a coordination among several individuals persist until more body of evidence comes along. In addition, it is important to note that “sequences of sequences” have not been examined in the present study, thus more complex levels of combinations are yet to be explored. In the Tyrrhenian Sea, bray call series showed a rate of 0.09 per minute, a value three-folds higher than the Strait of Sicily. The two study sites share two single element sequences (Sequence 1 and 10, possibly part of a more general repertoire shared at least among Mediterranean and Eastern Atlantic [59]), while multi-element sequences have been identified in the Tyrrhenian Sea only. The SDC index confirmed the high degree of dissimilarity between the bray-call repertoire of the two sub-populations. In cetacean species, it is known that local, ecological, and social specificities, such as group size, composition, and ecology may affect distribution and acoustic variations within populations (e.g., [60,61]). Here, bray-call differences in common bottlenose dolphins in the Tyrrhenian Sea and in the Strait of Sicily may have been shaped by such factors. In the Tyrrhenian Sea, several individuals show a high level of site fidelity [26]. Such a strong residency pattern seems not present in the Strait of Sicily, where a core of female shows site fidelity—even if lower than the Tyrrhenian sub-population—but a great number of individuals likely temporarily transient in the area [30,62]. Thus, the use of a more complex code of communication like bray-call sequences might be not useful to share information among individuals in this sub-population. In the Tyrrhenian Sea, photoidentification analysis revealed that resident dolphins (mostly females) have been observed in more than 65% of encounters with bray-call detection. Although common bottlenose dolphins are characterized by a fission-fusion social structure [63], they can establish long-term social relationships when inhabiting a specific area permanently. This phenomenon has been observed especially for females residing in estuary regions that coexist durably [64,65] (as in the Tyrrhenian Sea) and may explain the development of local communicative acoustic signals used to improve the group cohesion and facilitate individuals’ coordination. The presence of bray-call during social interactions [19] in an estuary area, and in feeding situations [24,25], seems to support their possible communicative role in both contexts. More specifically, [24,66] supposed that bray-call sequences may be used by common bottlenose dolphins to manipulate specific prey’s behavior and suggested that geographic differences in bray-call emissions may derive from a contextual learning process that is influenced by locally available prey. Unravelling the role of sequences might support discriminating different activity contexts, providing a new tool for studying habitat use and behavior through acoustic cues.

In this study, the acoustic parameters extracted from sequences and acoustic elements revealed variations between the two geographical areas when considering low frequency, high frequency, and element durations. Geographical variability in frequency and duration has been largely documented for other common bottlenose dolphin call types, such as whistles [60,67,68], and may has been developed as local adaptations to environmental variability [60,62,67,68,69,70]. Thus, studying the occurrence of these sequences could help in identifying different sub-populations and local specializations. Acoustic characterization of different populations and information sharing [71,72] have been proven to be useful for conservation, and this could be highly relevant for the common bottlenose dolphin in the Mediterranean Sea, a species listed in the Annex II of EU Habitats Directive (92/43/CEE).

## 5. Conclusions

In conclusion, this study proposed for the first time, quantification, depiction, and detailed description of common bottlenose dolphin bray-call sequences in the Mediterranean Sea, providing new and complete information about these vocalizations. Further research is needed to better understand their function(s) in feeding and social contexts (even extreme ones, like when a dead individual is present [73]), considering both their structural complexity and emission circumstances. In addition, due to the distinctiveness of the sequence structures, disruptions caused by anthropogenic (e.g., noise, fishery) and biological factors (e.g., prey type), as well as social and behavioral conditions, could be clearly revealed from the analysis of their patterns.

## Figures and Tables

**Table 1:** Survey platforms and methods for data collection, acoustic equipment, total recording effort and sampling periods for the two research sites.

Site	Platform and Survey Methods	Acoustic Equipment	Recording Effort	Year
Strait of Sicily	Boat-based survey using a motorboat powered by a four-stroke 100 HP outboard engine, in suitable weather conditions (sea state < 4 Douglas, wind force Beaufort < 4, no rain, no fog), at a steady speed of 6–8 kn. Both non-systematic haphazard (sensu [35]) and systematic sampling procedure. More details in [30,31].	One omnidirectional hydrophone Bruel e Kjer (Nærum, Denmark) model 8104 (sensitivity -205.6 dB re 1 V/1 μPa ± 4.0 dB), with a bandwidth < 0.1 Hz to >80 kHz) One digital sound card Avisoft Bioacoustics USGH 416HB (data format 16–24-bit WAV, sampling rate 44.1, 48 and 96 ks/s [36]).	3.8 h, resulting in 422 .wav files	2012–2015
Tyrrhenian Sea	Boat-based survey using a sailing vessel Beneteau Oceanis 41.1 powered by a 55 hp Volvo diesel engine, in suitable weather conditions (sea state < 3 Douglas, wind force Beaufort < 3, no rain, no fog), at a steady speed of 4–6 kn. Non-systematic haphazard sampling procedure (sensu [35]). More details in [26].	2017–2018: Two Colmar omnidirectional hydrophones (La Spezia, Italia) model GP0280 provided by CIBRA-Pavia University (sensitivity -168.8 dB re 1 V/μPa@ 5 kHz, flat frequency response from 1 to 30 kHz ± 5 dB), with a bandwidth 5 Hz–90 kHz 2019–2020: One towed hydrophone Aquarian Audio (Anacortes, WA, USA) model H1c-2018 provided by Nauta srl (sensitivity -199 dB re 1 V/μPa, flat frequency response from 20 Hz to 4 kHz ± 4 dB), with a bandwidth < 0.1 to >100 kHz. Digital sound card Roland Quad Capture UA55 (data format 16–24-bit WAV, sampling rate 44.1, 48 and 96 ks/s [36]).	115.3 h, resulting in 871 .wav files	2017–2020

**Table 2:** Definition of acoustic parameters measured for each bray call series and for each single element composing the sequence.

Parameter	Description
Element type	Type of acoustic element composing the sequence
Acoustic elements in the sequence (N)	Number of detectable elements composing each sequence
Sequence duration (s)	Time interval between the beginning of the first and the end of the last element
Element duration (s)	Duration of each element in the sequence
Inter-element interval (s)	Time interval between the end of an element and the beginning of the following one
Minimum Frequency (Hz)	The lower frequency of each element composing the sequence
Maximum Frequency (Hz)	The higher frequency of each element composing the sequence
Peak frequency (Hz)	The frequency with maximum amplitude in the spectrum (for POP and CR element types)

**Table 3:** Parameters measured for each bray call series and for each single element composing the sequence.

Element Type	Sequence Id Number	ID Element Type/Subtype (N of Elements)	Description
Gulp (GU)	1	GU1 (1711)	A single-element sequence composed of Gulp (GU1) repeated in series of 3 to 105 elements
	2	GU3 (233)–GU1 (229)	A multi-element sequence composed of 4 to 32 elements containing the repetition of Gulp pairs (GU3 and GU1)
	3	GU1 (97)–GU4 (93)	A multi-element sequence containing the repetition of Gulp pairs (GU1 and GU4), with 8 to 11 GU1 and 3 to 23 GU4 elements per sequence
	4	GU1 (108)–GU2 (97)–GU3 (107)	A multi-element sequence containing the repetition of Gulp triplets (GU1, GU2 and GU3), with the repetition of 2 to 22 GU1, 2 to 20 GU2 and 2 to 22 GU3 elements per sequence
Gulp (GU) and Pop (POP)	5	GU1 (32)–POP (412)	A multi-element sequence containing a series of POPs, alternated with 1 to 8 Gulp (GU1). The number of POP ranged from 9 to 27, while the number of POP series within the sequence varied from 1 to 16
Grunt (GR) and Gulp (GU)	6	GR1 (5)–GU1 (94)	A multi-element sequence containing a Grunt (GR1) followed by a series of 6 to 35 Gulp (GU1)
	7	GR1 (45)–GU1 (46)	A multi-element sequence containing a Grunt (GR1) and a Gulp (GU1) alternated, with 3–9 GR1 and 3 to 11 GU1 elements per sequence
	8	GR2 (20)–GU1 (142)	A multi-element sequence containing a Grunt (GR2) followed by a series of 3 to 26 Gulp (GU1)
Crack (CR), Grunt (GR) and Gulp (GU)	9	CR (157)–GR1 (34)–GU1 (34)	A multi-element sequence containing a series of 2–11 Cracks (CR) followed by a Grunt (GR1) and a Gulp (GU1)
Squeak (SQ)	10	SQ (61)	A single-element sequence composed of Squeaks (SQ) repeated in series of 2 to 14 elements
Squeak (SQ) and Gulp (GU)	11	SQ (47)–GU1 (41)	A multi-element sequence containing a Squeak (SQ) and a Gulp (GU1) alternated, with 2 to 13 SQ and 2 to 8 GU1 elements per sequence
	12	SQ (14)–GU1 (119)	A multi-element sequence containing a Squeak (SQ) followed by a series of 3 to 27 Gulps (GU1)
Low-Frequency Narrowband sounds (LNF) and Gulp (GU)	13	LFN (19)–GU1 (34)	A multi-element sequence containing a LFN and a Gulp (GU1) alternated, with 4–6 LFN and 3 to 17 GU1 elements per sequence

**Table 4:** Average values of the acoustic parameters of the 13 bray-call sequences, and of the element types and variants measured in each sequence.

Sequence Id Number	Sequence Typology	Element Type/Subtype	Sequence Duration (s)	Inter Element Interval (s)	Pair/Triplet Duration (s)	Inter-Pair /Triplet Interval (s)	Element Minimun Frequency (Hz)	Element Maximum Frequency (Hz)	Element Peak Frequency (Hz)	Element Duration (s)
1	Single element	GU1	6.3 ± 8.2 (0.15–115)	0.4 ± 0.3	-	-	293 ± 90	768 ± 154	-	0.039 ± 0.01
2	Multi-element organized in pairs	GU1	9.5 ± 10.4 (0.6–68)	0.11 ± 0	0.17 ± 0.05	0.44 ± 0.16	262 ± 49	753 ± 105	-	0.036 ± 0.01
		GU3					223 ± 49	572 ± 74	-	0.041 ± 0.01
3	Multi-element organized in pairs	GU1	7.9 ± 8.1 (1.4–36.4)	0.14 ± 0	0.25 ± 0.08	0.40 ± 0.10	290 ± 58	685 ± 76	-	0.038 ± 0.02
		GU4					592 ± 71	868 ± 86	-	0.070 ± 0.06
4	Multi-element organized in triplets	GU1	9.8 ± 8.3 (1.4–34.5)	0.13 ± 0.1	0.4 ± 0.11	0.23 ± 0.1	278 ± 40	775 ± 95	-	0.036 ± 0.01
		GU2					361 ± 83	584 ± 70	-	0.036 ± 0.01
		GU3					204 ± 35	491 ± 54	-	0.039 ± 0.00
5	Multi-element alternated	GU1	8.9 ± 7.4 (2–30)	0.2 ± 0 *	-	-	281 ± 52	842 ± 162	-	0.038 ± 0.01
		POP		-			-	-	813 ± 158	-
6	Multi-element	GR1	10.9 ± 8.4 (2–24)	0.45 ± 0.3 **	-	-	-	-	-	0.11 ± 0.03
		GU1					342 ± 76	874 ± 112	-	0.032 ± 0.00
7	Multi-element alternated	GR1	5.0 ± 4.6 (1.6–24)	0.17 ± 0.0	-	-	-	-	-	0.18 ± 0.10
		GU1					278 ± 64	700 ± 129	-	0.043 ± 0.01
8	Multi-element	GR2	4.7 ± 3.6 (1–18)	0.59 ± 0.4 ˟	-	-	-	-	-	0.14 ± 0.09
		GU1					324 ± 116	874 ± 234	-	0.043 ± 0.01
9	Multi-element	CR	4.9 ± 5.7 (0.6–26)	0.13 ± 0	-	-	-	-	1237 ± 279	-
		GR1		1.12 ± 0.4			-	-	-	0.14 ± 0.09
		GU1		1.25 ± 0.5			395 ± 243	750 ± 238	-	0.030 ± 0.01
10	Single element	SQ	3.7 ± 1.9 (0.6–8.3)	0.5 ± 0.3	-	-	535 ± 204	892 ± 303	-	0.17 ± 0.07
11	Multi-element alternated	SQ	5.5 ± 3.9 (1.4–15.9)	0.65 ± 0.2	-	-	472 ± 120	772 ± 172	-	0.13 ± 0.07
		GU1					306 ± 114	705 ± 114	-	0.044 ± 0.01
12	Multi-element	SQ	5.5 ± 3.9 (1.4–15.9)	0.3 ± 0.1 ˟˟	-	-	303 ± 39	621 ± 99	-	0.20 ± 0.08
		GU1					326 ± 136	829 ± 220	-	0.039 ± 0.01
13	Multi-element alternated	LFN	4.4 ± 2.7 (1.6–7.9)	0.3 ± 0.2	-	-	256 ± 64	650 ± 97	-	0.084 ± 0.04
		GU1					246 ± 58	533 ± 151	-	0.035 ± 0.00

* average period between the last POP and the following GU1; ** average period between GR1 and the first GU1; ˟ average period between GR2 and the first GU1; ˟˟ average period between SQ and the first GU.

## Data Availability

The data presented in this study are available to any qualified researcher on request from the corresponding author.
